# An Unusual Case of ST-Elevation Myocardial Infarction (STEMI) Mimicker: Pneumopericardium Secondary to Gastro-Pericardial Fistula

**DOI:** 10.7759/cureus.39358

**Published:** 2023-05-22

**Authors:** Asmaa Ahmed, Niraj Neupane, Devesh Rai, Cameron Hall

**Affiliations:** 1 Internal Medicine, Rochester Regional Health, Rochester, USA; 2 Cardiology, Rochester Regional Health, Rochester, USA

**Keywords:** chest pain, gastric cancer, gastro-pericardial fistula, stemi mimickers, pneumopericardium

## Abstract

Pneumopericardium is defined as the collection of air inside the pericardium. Gastro-pericardial fistula is one of its rarest etiologies. We are presenting a case of pneumopericardium due to gastro-pericardial fistula secondary to gastric cancer presented with an inferior ST-elevation myocardial infarction (STEMI)-like picture.

Our case is a 57-year-old male with a past medical history of metastatic gastric cancer status post chemotherapy and radiotherapy who presented to the emergency with acute onset severe burning chest pain with radiation to his back. He was diaphoretic, saturating 96% on room air, and hypotensive with a blood pressure of 80/50 mmHg, and his EKG showed sinus rhythm with a heart rate of 60 BPM and ST elevation in inferior leads meeting STEMI criteria.

The patient was transferred for an emergency coronary angiogram with possible percutaneous intervention. Surprisingly, no significant lesions in his epicardial vessels would corroborate his clinical presentation and EKG changes. The decision was to obtain CT angiography to exclude aortic dissection and pulmonary embolism. His CT chest revealed a large pneumopericardium with a gastric-pericardial fistula.

A nasogastric tube was placed with suctioning of gastric contents. Given his tamponade physiology, it was decided to do emergent pericardiocentesis draining only 20 cc of gastric contents and a significant amount of air. After the procedure, the patient was transferred to the ICU with stable hemodynamics. The case was discussed with surgery, but given his inoperable cancer, a palliative team was involved. Acknowledging his very poor prognosis, the patient requested discharge to home with home hospice.

As reported in the literature, pneumopericardium is rare, and gastro-pericardial fistula associated with gastric cancer is even rarer. Clinical presentation is variable and can be confusing. Providers should be aware of how a patient with gastric cancer can be complicated with pneumopericardium, and they should have a lower threshold of suspicion in patients having risk factors. CT scan is the most sensitive tool for diagnosis.

## Introduction

Pneumopericardium was defined as the collection of air inside the pericardium by Britcheteau et al. in 1844 [1]. Most commonly, pneumopericardium is caused by trauma, representing 60% of cases [2]. It can also be iatrogenic or spontaneously secondary to infections, including tuberculosis and human immunodeficiency virus disease, cocaine inhalation, and fistula communication between the pericardium and other structures [2,3]. Gastro-pericardial fistula is one of the rarest etiologies of pneumopericardium, with very high morbidity and mortality [3]. Gastro-pericardial fistulas are most commonly associated with previous surgery as Nissen fundoplication, reflux esophagitis, and gastric cancer with a range of presentations including dyspnea, chest pain, epigastric pain, fever, and up to cardiac tamponade and arrest [3,4]. We are presenting a case of pneumopericardium due to gastro-pericardial fistula secondary to gastric cancer presented with an inferior ST-elevation myocardial infarction (STEMI)-like picture.

## Case presentation

**Figure 1:**
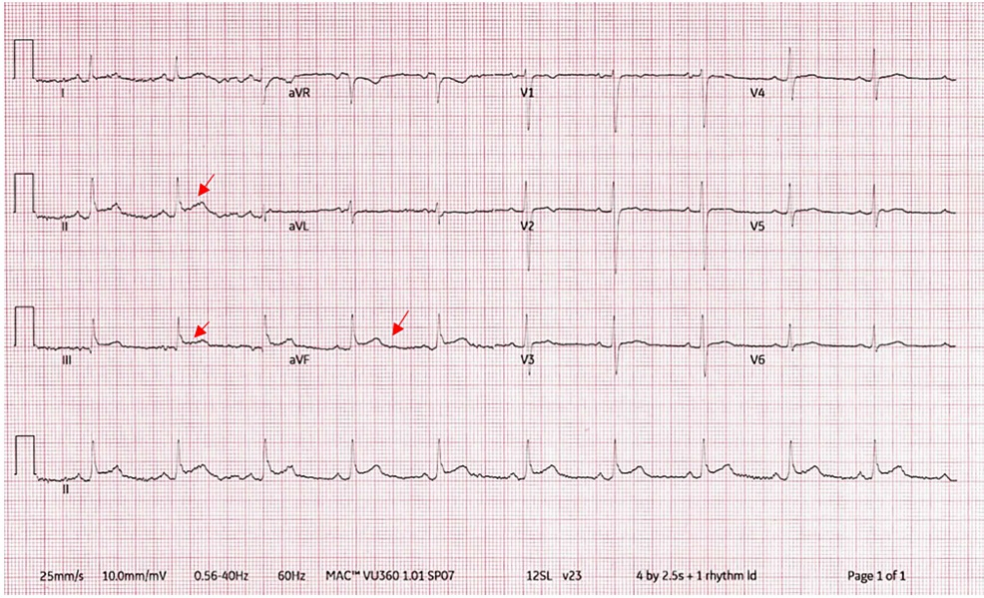
Surface EKG on presentation showing ST elevation in inferior leads

A 57-year-old male with a past medical history of metastatic gastric cancer status post chemotherapy and radiotherapy, Lynch syndrome, and colon cancer since 2007 status post surgery and chemotherapy presented to the emergency department with acute onset severe chest pain that he describes as burning in nature with radiation to his back. He was notably in extremis, could not get comfortable on the gurney, and was diaphoretic. He was saturating 96% on room air, hypotensive with a blood pressure of 80/50 mmHg, and his EKG showed sinus rhythm with a heart rate of 60 BPM and ST elevation in inferior leads meeting STEMI criteria (Figure 1) [5].

The patient was diagnosed with inferior STEMI and was immediately transferred for an emergency coronary angiogram with possible percutaneous intervention. Due to his restless nature, inability to lay flat, and severe pain, the patient required intubation which was completed emergently in the cardiac catheterization lab (cathlab).

Surprisingly, no significant lesions in his epicardial vessels would corroborate his clinical presentation and EKG changes. Interestingly, during fluoroscopy, flashes of pericardial air were appreciated. A stat bedside echocardiogram showed mildly impaired left ventricular systolic function with an ejection fraction of 45-50% but with poor windows due to a significant amount of air.

**Figure 2:**
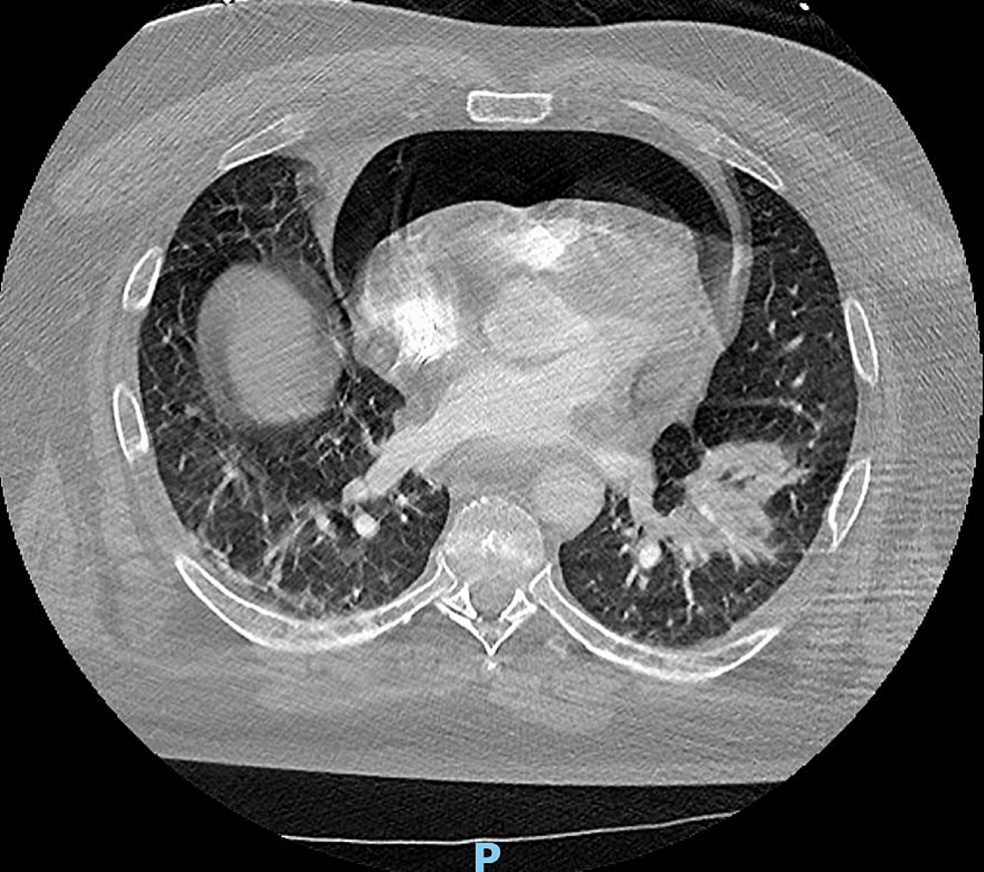
CT scan showing air around the heart (pneumopericardium)

**Figure 3:**
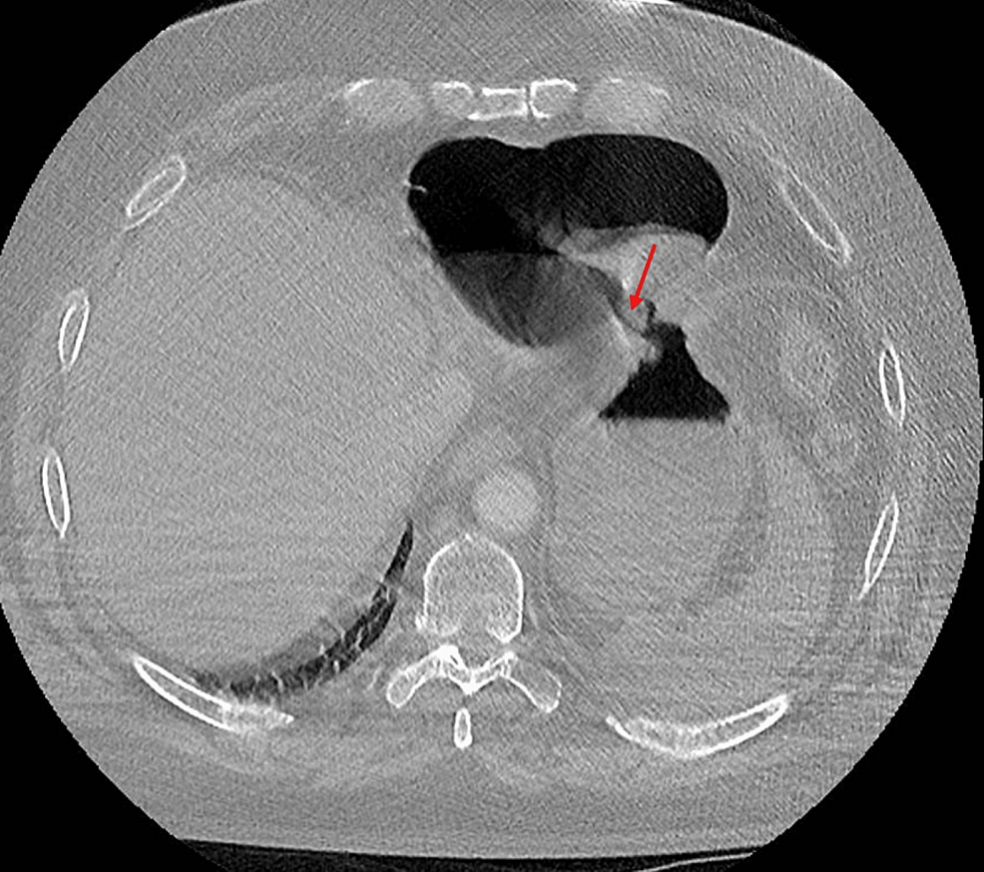
CT scan showing a connection between the stomach and the pericardium (arrow referring to the gastro-pericardial fistula)

Due to these constellations of findings, it was decided to obtain CT angiography to exclude aortic dissection and pulmonary embolism (PE) and investigated the source of pericardial air seen during coronary angiography. His CT chest didn't detect any PE or aortic dissection but revealed a large pneumo-pericardium with a gastric-pericardial fistula (Figures 2-3).

**Figure 4:**
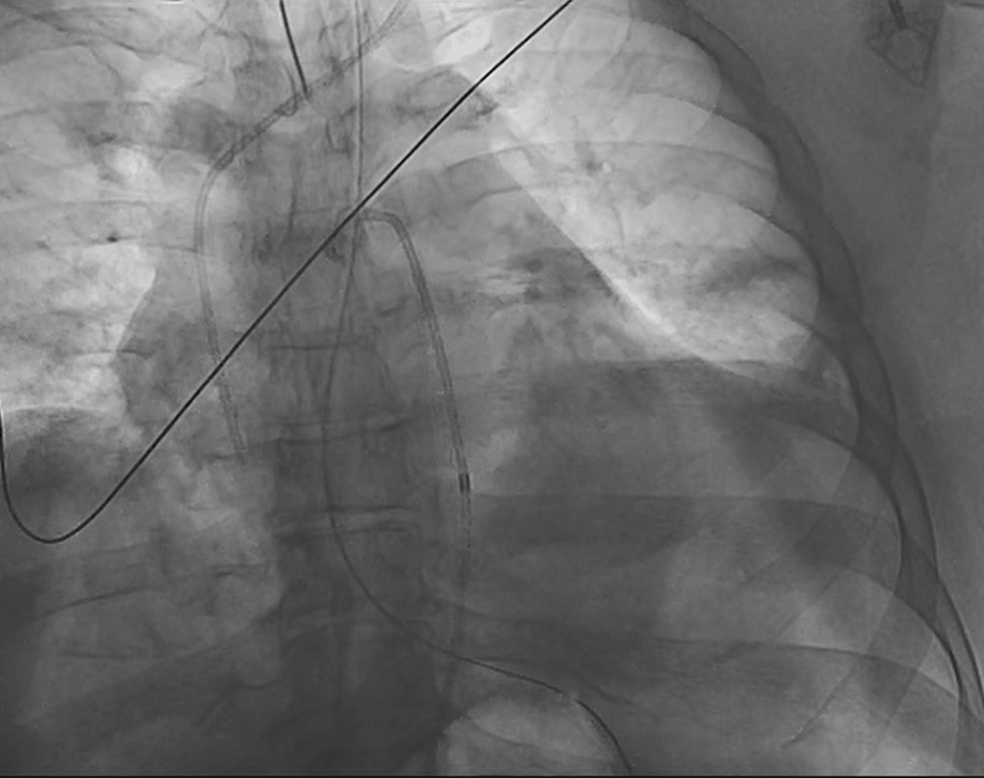
Cathlab image showing the heart after pneumopericardium drainage

**Figure 5:**
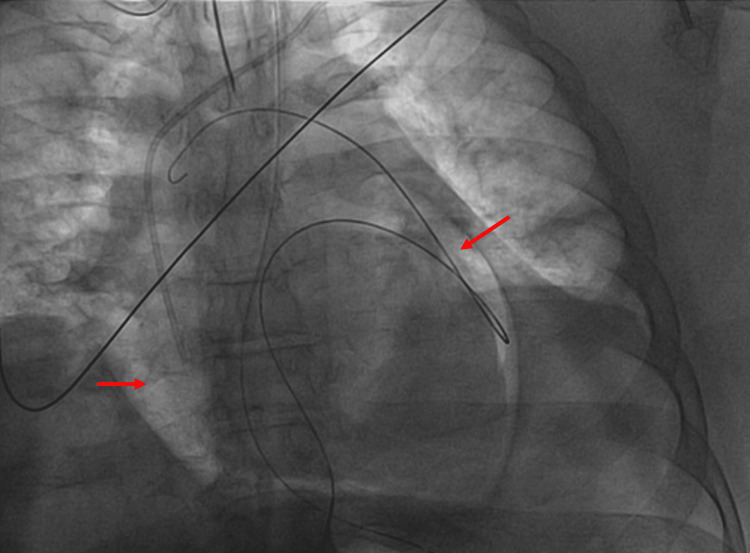
Cathlab image showing pneumopericardium before draining (red arrows referring to the air around the heart)

A nasogastric tube was placed, and 250 mL of gastric contents were drained after suctioning. The case was discussed with thoracic and abdominal surgery, but given his inoperable cancer and poor prognosis from the malignancy, there was no plan for surgery at the time. Given his ongoing tamponade physiology, it was decided to pursue emergent drainage of the pericardial collection. He was returned to the cardiac cathlab, and a successful subxiphoid pericardiocentesis was performed, draining 20 cc of gastric contents and a significant amount of air (Figures 4-5).

With these interventions, his hemodynamics stabilized. The patient was transferred to the ICU for further management.

Once in the ICU, he was successfully extubated which facilitated goals of care discussion with the patient and his family. The palliative team was intimately involved, providing guidance in analgesia and goals of care discussions. Acknowledging his very poor prognosis, the patient requested discharge to home with home hospice.

## Discussion

As Bollak and Brady mentioned [5], there are many entities that can cause ST elevation and, thus, mimic STEMI with no coronary plaque rupture or coronary occlusion. These STEMI mimickers include myopericarditis, aortic dissection, early repolarization, ventricular aneurysm, Takotsubo cardiomyopathy, Brugada syndrome, hyperkalemia, hypothermia, and hypercalcemia.

Our patient was not hypothermic and didn't have significant electrolyte abnormalities. His clinical presentation was not compatible with the benign early repolarization or with Brugada syndrome. In addition, with no aneurysm and no segmental dilatation, his echocardiogram strongly argued against ventricular aneurysm and Takotsubo cardiomyopathy. Given the chest pain and concerning EKG changes, differentials that should be investigated would be inferior STEMI, PE, and aortic dissection. Another differential would be acute pericarditis, especially with PR elevation and ST depression in aVR in his EKG. Based on that, transferring the patient to the cathlab with an initial STEMI diagnosis, as well as getting a CT afterward to exclude the other two fatal differentials, PE and aortic dissection, was justified. Likewise, in our clinical presentation, it is likely pneumatic compression of the RCA from tamponade best explained the EKG changes as these resolved once the pericardial drain was placed.

A few cases of pneumopericardium presenting with STEMI mimicking pictures have been reported in the literature. Regardless of the localization of the ST elevation in the initial EKG, these cases were eventually diagnosed using a very similar approach involving a coronary angiogram followed by a CT scan. The diagnosis of pneumopericardium was done retrospectively by radiographic imaging studies in most of the literature-reported cases [1,6-13].

Based on that, we agree that understanding the presentation of pneumopericardium and considering a lower threshold for diagnosis, especially in at-risk patients, can be extremely helpful in avoiding unnecessary investigations, especially if they will lead to invasive procedures.

Different presentations have been identified for pneumopericardium in literature. Chest pain or shoulder pain is the most commonly reported presentation, followed by dyspnea [4]. Hirani et al. identified 11 cases of pneumopericardium secondary to malignancy, and most of the identified were related to cancer lung and cancer esophagus [14]. Prior gastro-pericardial surgery was most commonly described as a cause of gastro-pericardial fistula, while a history of gastric cancer is considered very rare [3]. Of the 65 cases of gastro-pericardial fistula identified by Azzu et al., only six cases were caused by cancer perforation [4]. The history of chemotherapy and radiation therapy was also reported as a cause of gastro-pericardial fistula due to the toxic effect on the endothelium with the potential adhesion leading to fistula formation [3]. Rathur et al. reported a case of gastro-pericardial fistula with a history of chemotherapy for metastatic colon cancer with no evidence of gastric dysplasia or malignancy [3]. Our case had a history of gastric cancer with chemotherapy and radiation therapy and metastatic colon cancer. With these risk factors in mind, there should be a lower threshold for suspicion of gastro-pericardial fistula and pneumopericardium.

Pneumopericardium is most commonly diagnosed with a chest X-ray (CXR), CT scan, and echocardiogram [14]. While CXR can diagnose pneumopericardium by the presence of a radiolucent halo surrounding the heart silhouette, a CT scan can also show underlying cancer, metastasis, and communication between the stomach and the pericardium [14]. Echocardiography also plays a vital role in diagnosing and assessing cardiac function and the degree of cardiac compromise with tamponade. However, the large amount of air can blunt the probe images making the diagnosis challenging [14]. In some patients, transesophageal echocardiography may be warranted. In our case, the diagnosis was confirmed retrospectively by a CT scan after emergent coronary angiography. Again, suspecting pneumopericardium in such cases and proceeding with early radiographic imaging such as CXR or CT can save the patients from unnecessary invasive interventions such as coronary angiography, which puts the patients at an unnecessarily higher risk of complications.

## Conclusions

Pneumopericardium is rare, and gastro-pericardial fistula associated with gastric cancer is even rarer. Clinical presentation is variable and can be confusing. However, gastro-pericardial fistula complicated with pneumopericardium should be suspected in patients having risk factors. A CT scan is the most sensitive tool for the diagnosis of pneumopericardium and the fistula.
